# The christian religious education teachers’ attitudes toward the five-stage lesson plan framework in Kenya: A gender-based analysis

**DOI:** 10.1016/j.heliyon.2023.e19104

**Published:** 2023-08-11

**Authors:** Victor O. Saoke, Collins M. Musafiri, Zachary N. Ndwiga, Pauline W. Githaiga

**Affiliations:** a Department of Education, University of Embu, PO BOX 6-60100, Embu, Kenya; b Cortile Scientific Limited, PO Box 34991-00100, Nairobi, Kenya; c Department of Curriculum, Instruction and Educational Management, Egerton University, PO BOX 536, Egerton-Njoro, Kenya

**Keywords:** Gender differences, Teachers' attitude, Five-stage lesson plan framework

## Abstract

New approaches have emerged in teaching Christian Religious Education (CRE). Five-stage lesson plan framework (FSLP) is one of the innovative approaches applicable in all learning areas. Even though the framework is learner-oriented, understanding teachers' attitudes towards the framework are essential. Developing a positive attitude among the stakeholders remains crucial in implementing any teaching approach. Therefore, this study assessed gender differences in the attitude toward the five-stage lesson plan framework among CRE teachers in Meru County, Kenya. A multi-stage sampling technique randomly selected 143 and 83 female and male CRE teachers. Using a semi-structured questionnaire, quantitative data were collected in a cross-sectional survey. Chi-square and *t*-test statistics were applied to test the statistical significance of the dummy and mean value of continuous variables using sex-disaggregated data. The study revealed that teachers' attitudes towards the FSLP framework did not significantly differ between male and female teachers. The study employed multiple linear regression modeling to assess the determinants of the attitudes towards the five-stage lesson plan framework for male, female, and pooled teachers. Similar determinants influenced teachers' attitudes towards the FSLP framework: academic qualification, teaching experience, utilization, and awareness. Researchers established that educational qualification was a negative and significant determinant of attitudes towards the FSLP framework among the participants. Teaching experience, awareness, and utilization of the FSLP framework among CRE teachers were positive and significant determinants of the attitudes towards the FSLP framework. The study findings implied development of policies inclined to the attitudes supporting the proper implementation of newly emerged teaching strategies among the stakeholders. The supplement schemes should be restructured and shaped to meet teachers' specific needs and preferences regarding the utilization of educational innovations to enable CRE teachers to select and practice the FSLP framework.

## 1 Introduction

Teaching is a demanding occupation globally [1]. The current education system does not meet the modern young generation's spiritual and instructional needs marked by continuous change [2]. At a young age, they experience the difficulty of the contemporary world and occasionally appear conflicting to the teachings of Religious Education. The role of Religious Education is to enable learners to understand the nature of the present secular and pluralistic society [2]. New approaches have emerged in teaching across the learning areas [3]. The new techniques meant transitioning from a teacher-oriented approach to student-oriented teaching [4]. The new methods need continuous enhancement to support learners' improvement on the recognized teaching objectives more efficiently than the distinctive content delivery approaches in a classroom setting [5].

Innovative instructional approaches are a significant concern in CRE but still limited among teachers [6]. Even though changes have occurred in teaching, CRE still leans toward traditional learning methods [3]. Christian Religious Education teachers are reduced to preachers because they do not employ proper strategies for sequential learning [7]. Nevertheless, new and advanced teaching approaches renovate traditional teaching to improve the understanding and application of new knowledge under delivery [8]. The innovative approaches are primarily learner-centered [9]. Teachers' innovativeness creates effective teaching and learning that increases learners' creativity, mainly in today's information society [10]. This study offers an innovative pedagogical framework known as the five-stage lesson plan (FSLP) based on differentiated learning styles to improve CRE pedagogy.

The FSLP framework is an innovative teaching approach that helps learners link new ideas to familiar ideas, arouse curiosity, and create interest during the learning process [11]. It provides a supportive climate, discourse features, cognitive activation, and instructional clarity during the content delivery [12]. The framework was progressively developed from Herbartian and Madeline Hunter's approaches [13], aiming at learners' preparation to understand the new ideas. Initially, Herbartian and Madeline's ideas suggested seven steps for the framework. However, their followers reformed the suggested steps to five stages of the FSLP framework, a concept of educational teaching relevant to the divergent classroom today [14]. This aimed to make the framework simple and less time-consuming in its full implementation. The five stages, anticipatory set, the introduction of new materials, guided practice, independent practice, and the lesson closure, must be implemented jointly to ensure sequential content delivery [15]. The framework is based on fundamental assumptions and philosophies that all learners can improve academically [16]. It ensures flexible language diversity suitable to learners' instructional needs [17].

The efficiency of educational innovations, including the FSLP framework, is highly influenced by the implementers' attitudes [18]. Therefore, teachers' attitude remains crucial in implementing the FSLP framework. A teacher can adopt innovations if a positive attitude is developed [19]. A positive attitude makes teaching more operative and jobs rewarding, while a negative perception makes the teaching unpleasant and tedious [20]. Attitude has three components: the situation, the perceiver, and the target [21]. In this study, CRE teachers represent the perceiver; attitude on the FSLP framework is the target while teaching is the situation. Therefore, the need to assess the attitude toward the FSLP framework among CRE teachers.

Even though teaching CRE is the situation in this study, teachers and learners, both male and female, perceive the subject negatively, making its teaching challenging [22]. The negativity toward CRE worsened through a proposal by the Ministry of Educational sector that the government would only support higher learning courses relevant to the 2030 vision developmental goals [23]. Christian Religious Education was not a pre-requisite for entry to highly classified professional courses in terms of ranking like law, computer science, engineering, and medicine. As a result, the Kenyan education sector is presently employing changes to achieve efficiency and effectiveness of meaningful learning in all the learning areas [24]. Implementing these changes has been changed from the school management to teachers [25]. For these implementations to succeed, both male and female, teachers must develop positive attitudes towards these changes [21].

Researchers frequently use the term sex when referring to differences between male and female students' or teachers' educational experiences [26]. Various studies exploring the effect of demographical variables such as gender, experience, and academic qualifications on the attitude of learners and teachers towards newly emerged strategies have been carried out [27; 22; 2328]. Generally, attitude is a social paradigm affected by various aspects such as gender, experience, and academic qualification [21]. About the mentioned variables, the study analyzed gender as one of the critical factors influencing CRE teachers' attitudes toward the FSLP framework. It is a dominant element capable of influencing the attitude of any teacher in the profession [27]. In West Java, Indonesia, a study was conducted on gender as a determining factor of attitudes toward information related to a person's medical history [28]. The findings outlined that gender differences do not affect attitudes towards health information.

Contrary to the findings, a study assessed teachers' attitudes towards instructional practices [29]. This study outlined a significant variation in the attitude of female and male instructors towards the teaching profession. Male instructors were less optimistic than their counterparts in their attitude towards teaching. In agreement with the study findings [30], in her study confirmed a substantial dissimilarity in the attitude of female and male instructors towards their instructional practices.

Although a newly formed underlying set of facts has been established on teachers' planning practices and skills, the gender of the teacher can significantly influence attitudes towards the framework. Gender plays a crucial role in understanding the differences in attitude towards the usefulness of newly emerged strategies and ease of use among the stakeholders [31]. Additionally, a study is significant if it includes attitude, awareness, and both genders as variables [32]. Thus, the study was significant as it involved the variables mentioned above. Unfavorable policies are significant hurdles to the CRE transformation and modernization, religious plurality, secularity, and unbendable competition from other disciplines [33]. The subject has not been given attention in Kenyan secondary schools [34]. However, researchers and educationists have advocated for enhanced techniques and teaching CRE based on real-life experience and learner-centered approaches [35]. Hence, new forms of teaching CRE have emerged, such as the FSLP framework and life approach skills. Teachers must apply this framework to enable learners to appreciate the content. However, the gendered differences in the attitude toward the FSLP framework among the CRE teachers could play a central role in defining the teachers' perspectives on the framework.

Although various concerns are covered, specific issues have been considered frequently. For example, the impact of gender on teachers' attitudes towards educational innovation and the teaching profession at large. Except for the literature diversity, investigation based on the gendered differences in the attitudes towards newly emerged teaching strategies in social sciences appears to be rare. Additionally, studies on gender position in innovative teaching approaches such as the FSLP framework, support system, and policy in CRE are inadequate [36]. What bearing the gender has on the attitude of CRE teachers toward new teaching strategies to throw light on this study was conducted to assess the gendered differences in the attitudes towards the FSLP framework. The studies cited elaborately indicate the role of gender as a determinant of attitude towards the teaching profession and health information. There is, however, little or no mention of the role of gender in determining attitude towards the FSLP framework in teaching CRE.

Even though the FSLP framework is learner-oriented and applicable in all learning areas, there is limited information on gendered differences in its attitude among CRE teachers in Kenya. Past literature highlights teachers' attitudes toward the newly emerged approaches to teaching [20]. Despite the importance of the FSLP framework in education, there have been no or scanty studies on gendered differences in teachers’ attitudes towards the framework. Therefore, the study intended to answer the following research questions; i) what are the gendered differences in attitude toward the FSLP framework, and ii) what are the gendered determinants of attitude toward the FSLP framework among CRE teachers in Meru County, Kenya.

### 1.1 Theoretical background

Several theories, such as social learning theory [37], neurophysiological bases theory [38], self-perception theory [39], cognitive developmental theory [40], gender schema theory [[41,42]], and the triadic of attitude formation theory [[43,44]], could be used to describe gender differences and determinants of the attitudes toward the five-stage lesson plan framework among CRE teachers. The study employed self-perfection theory, gender schema theory, and the triadic of attitude formation theory as the researchers' key theoretical framework in this study, similar to [[[45], [46], [47], [48]]]. The theories are explaining the effects of teachers' awareness, utilization, experiences and qualification on their attitudes towards the innovative teaching strategies as indicated in the gender-based analysis by Ref. [45]. Originally, self-perfection theory focused on the teachers' attitude towards teaching innovation. However [49], speculate that literature applies this theory to the teachers' practices by considering gender differences on attitude based on teachers’ qualification and experience. Self-perfection theory proposes that teachers, male or female, differ in attitude frames toward teaching innovation strategies and that the teaching results are significantly influenced by these attitudinal differences [50].

Subsequently, it is challenging to measure the gender attitudinal frames of the teachers, the literature uses visible characteristics of teachers, such as gender, awareness, utilization, experience and qualification as substitutions for attitudinal edges [39]. [47] illustrates teachers' gender attitudinal frames as their thoughtful processes of assessing and acquiring information. He additionally argued that teachers’ thinking edges are linked to their morals, awareness, knowledge, experience, and dispositions and extremely impact how they pursue and understand facts. Thus, this suggestively impacts the policymaking process, hereafter the teaching results.

Gender schema theory accepts the unfavorable effect of attitude on teachers' application of novel teaching strategy [42]. It clarifies how teachers apply prominent demographics such as gender, experience, awareness and qualification to group themselves into different social categories [28]. Considering this process, teachers shape self-worth and communal uniqueness by segregating themselves as associates of a particular group and paralleling themselves with other clusters [51]. Teachers categorizing themselves could lead to gender attitudinal favoritisms. Teachers' composition in gender distribution, awareness, utilization, qualification and experience variety are vital as it influences the teachers’ decision-making process towards the innovative strategy such as the five-stage lesson plan framework [52].

The triadic model of attitude formation shaped the theoretical base of this study [[43,44]]. The theory has three elements; affective, behavioral, and cognitive. Choice of the FSLP framework among CRE teachers is affected by many dynamics of the education scheme such as experience, awareness and qualification [44]. According to Ref. [44], attitudinal changes in a single component combine to create a scheme and are inter-reliant. Therefore, CRE teachers' choice of the FSLP framework is a product of changes in other elements. Cognitive and affective elements of attitude are closely related. The affective element incorporates the teacher's positive or negative emotions about the FSLP framework. The behavior element comprises the purpose to react in a certain way preferring the FSLP framework based on its utilization and teachers' awareness as described by Ref. [28]. The cognitive element mentions the thoughts and beliefs a teacher holds about the object of preference, the FSLP framework. Christian Religious Education teachers may therefore view the choice of the FSLP framework as a beneficial area, and therefore this perspective contributes to the establishment of preference.

For example, a CRE teacher's inclination to select the FSLP framework might entail an emotional reaction (the affective element) designed to express a positive effect. The intent is to select the FSLP framework as the instructional approach (the behavior element) and believe that content delivery in CRE using the FSLP framework is beneficial to the teacher and their learners (the cognitive element). Teachers' favorite teaching approach is an imaginary hypothesis that cannot be witnessed straightly. The reality of such a hypothesis can be drawn from the teacher's actions (The purpose of selecting the FSLP framework). The behavior can be based on the effects of curricula and environmental-related factors. The triadic model of attitude formation theory advocates that considering gender, experience and qualification diversity is sufficient to make a significant impact on the choice of innovative strategies of teaching.

## 2 Methodology

### 2.1 Study area

The study was carried out in Imenti South, Imenti North, Imenti Central, Igembe East, Igembe West, and Buuri sub-counties respectively, Meru County, Eastern Kenya. These sub-counties had 59, 66, 53, 63, 69, and 36 secondary schools. Meru County had 367 secondary schools during this research study. The study involved all the 24 private and 307 public secondary schools in the mentioned sub-counties, thus a census study was adopted. This is because Buuri sub-County supported the pilot study of the research instruments hence not involved in the data collection process. During the study, Meru County had 223, 83, 21, and 4 Sub County, county, extra county, and national secondary schools respectively. Out of 6520 secondary school teachers in Meru County, 646 were CRE teachers. The sub-counties had 125, 140, 102, 131, and 148 CRE teachers respectively.

### 2.2 Study variables description

This study's dependent variable was the CRE teachers' attitude towards the FSLP framework. A five-point Likert scale applicable to the attitude items where each item had five alternatives, including strongly disagree (SD), disagree (D), undecided (D), agree (A), and strongly agree (A). 5 was (SA), 4 (A), 3 (U), 2 (D) or 1 (SD) as indicated by [ [32,53,54]].

The study's independent variables were selected based on the ability to predict attitudes toward a teaching innovation strategy such as the FSLP framework [55]. They were grounded on the ability to envisage attitudes toward the FSLP framework, as specified by the previous literatures [ [56,57]]. Gender, academic qualification, teaching experience, awareness, and utilization are the clarifying aspects of the remarkable attitudes to the FSLP framework [ [28,58]]. Gender is male or female, an explanatory variable that could distinguish participants in this study [59]. Gender schema theory, self-perfection theory and the triadic model theory, both describe teachers' awareness, qualifications, utilization and experience as the main determinants of attitudes towards an instructional method [ [39,42,44]]. Gender differences in the educational field have distinguished male and female teachers in classroom behavior and teaching practices, such as applying the FSLP framework [60]. Literature on gender differences has described it as an element capable of manipulating teachers' attitudes in the field [ [[27], [28], [29], [30]]]. Thus, the investigation attempted to determine the gendered differences in attitudes toward the FSLP framework among CRE teachers.

Academic qualification, as a variable, is described as the CRE teachers' highest level of education. It is directly associated with teachers' attitudes towards teaching in the profession [61]. Teachers' qualifications are considered a significant predictor of their attitudes towards teaching in innovation implementation in schools [62]. Therefore, this study considered teachers' qualifications in predicting their attitude towards the FSLP framework.

Teaching experience is described as the number of years a teacher has taught. It is essential to effective classroom performance [63]. When a teacher is introduced to a new pedagogical technique, such as the FSLP framework, it is customary to develop a set of attitudes towards that teaching approach directed by their experience and the required qualification during the introduction process [64]. Therefore, the study considered teaching experience a predictor variable in establishing the attitude toward the FSLP framework.

A key aspect underlying a CRE teacher's attitudes to innovation, such as the FSLP framework, is their level of awareness and understanding of the framework. Awareness is described as the number of the FSLP framework stages a teacher was aware of. Creating awareness of an approach among teachers increases the positive attitudes towards the same approach [65]. Therefore the need to identify CRE teachers' awareness of attitude toward the FSLP framework.

Lastly, utilization of the framework is described as the number of the FSLP stages implemented by the teacher. Continuous practicing of new teaching approaches, such as the FSLP framework, increases the positive attitudes toward the approach [66]. Therefore the need to establish the utilization of the framework in collaboration with the attitude towards the framework. According to Burgess (1986), attitude is a social factor influenced by many aspects, such as gender, experience, qualification, awareness, and utilization of the framework among the stakeholders. Therefore, all the mentioned variables formed part of the clarifying aspects of the remarkable attitudes to the FSLP framework.

### 2.3 Sampling procedure and sample size

A multi-stage sampling procedure for CRE teachers and a cross-sectional survey were employed similar to Ref. [67]. First, Meru County was chosen due to poor performance and a negative attitude towards teaching CRE [68]. Secondly, purposive sampling of five sub-counties, i.e., Imenti Central, Imenti North, Imenti South, Igembe East, and Igembe West, from the six total sub-counties, including Buuri in Meru County, was done. The number informed secondary schools and CRE teachers. Third, an entire sampling procedure was employed to collect data from the five sub-counties. A proportionate to size sampling procedure was used in determining CRE teachers sampled per sub-county. Lastly, a random sampling procedure was used to collect data from 247 CRE teachers in the five sub-counties. 39, 48, 54, 50, and 56 CRE teachers were sampled from Imenti Central, Imenti South, Imenti North, Igembe East, and Igembe West sub-counties respectively. The population was 646 CRE teachers in the five sub-counties (Ministry of Education, Meru County). The participants' sample size was determined using Slovin's formula [69] as described by Ref. [67].

Researchers calculated the sample size as described in equation (1).

(1) n=N/(1+Ne²)

where n is the sample size, N is the total population (646), and e is the margin of error (0.05 margin of error). Therefore, the sample size was 247. However, this study's response rate was 91%, with 226 CRE teachers.

### 2.4 Data collection instrument

A semi-structured questionnaire was used for data collection. A semi structured questionnaire has both open and closed ended questions [70]. The questionnaire was appropriate for this study since it allowed the investigator to obtain opinions and views from the participant [64]. The questionnaire administration involved 247 CRE teachers with the handy observation by the researcher. It had questions on attitudes toward the five-stage lesson plan framework, CRE teachers' background, and explanatory variables. The CRE teachers were called upon to voluntary consent before contributing to the study. Researchers carried out a pilot to evaluate the appropriateness of the research instrument to collect dependable and feasible data in line with the research objective.

### 2.5 Statistical analysis

Data were analyzed using STATA 15.0 software. Data cleaning and coding were executed before statistical analysis. Researchers performed descriptive statistics comprising percentage, standard deviation, standard error, means, and inferential statistics multiple linear regression. In this study, Researchers used correlation analysis for the validity of the research questionnaire. Justification of the questionnaire's validity was based on the inspection by the researcher's supervisors and specialists in the research field—this enriched modification of the research questionnaire to enhance its validity. Therefore, the questionnaire content validation was suitable for establishing the level to which the set constructs provided a relevant and representative sample of the tasks under study [71].

Generally, piloting aims at inspecting the constructs in a questionnaire to establish whichever ambiguities and uncertainties are before it is employed in a study [72]. The CRE teachers' questionnaire was piloted for validity and reliability. Researchers employ a minimum of 12 observations to construct a confidence interval [73]. According to Ref. [74], at least 12 subjects cluster should be considered for pilot studies in the medical field. 12 CRE teachers from 6 secondary schools in the Buuri sub-county, Meru County, were involved in piloting the questionnaire. The validity of the instrument was tested by subjecting the variables score (nine attitude constructs) and the total score to the Pearson's Moment correlation in STATA. The reliability was tested using the Cronbach Alpha formula. The formula is shown in equation (2);

(2) α=K.c/[v+(K–1)c]

where.

K is the number of items in the test tool

c is the mean inter-item covariance among the items

v is the overall mean-variance.

The formula was appropriate since the questionnaire was administered once and had close-ended multiple-choice constructs. The formula is applicable when assessing the reliability of polychotomous constructs [75]. Based on the p values (p < 0.05) it can be concluded that the nine items were valid. The study questionnaire had a reliability coefficient of 0.738. It was consistent since its coefficient was above the 0.7 thresholds.

Researchers subjected the constructs for the attitudes of CRE teachers towards the FSLP framework to Cronbach Alpha, as indicated by [ [67,[76], [77], [78]]]. It is a significant concept in evaluating questionnaires and assessments [79]. Researchers verified the validity of the constructs on the attitude towards the FSLP framework. Researchers then calculated the weighted average index (WAI) to rank attitudes toward CRE teachers' five-stage lesson plan framework. The study adopted research by [ [80,81]] as described in equation (3).

(3) $$WAI=\frac{\sum sd+d+u+a+sa}{N}$$

where *WAI* is the weighted average index, sd is strongly disagree, *d* is disagree, u is undecided, a is agree, sa is strongly agree.

Researchers examined data fitness and quality using diagnostics tests, including the Cronbach test for reliability. Descriptive characteristics were categorized into males and females. Cross tabulation (Chi-square) analysis was executed on categorical variables and a *t*-test on continuous variables to determine whether there was an association between gender and the explanatory variables. Panel vector autoregression (VAR), system Generalized Method of Moments (GMM), and Ordinary Least Squares (OLS) model are also models suitable for our study. However, this study had many explanatory variables. Therefore, the study required a statistical approach to describe multiple associations of several variables with one continuous outcome. Notably, the gendered attitudes toward the FSLP framework could also differ based on participants' characteristics, including; academic qualification, teaching experience, awareness, and utilization of the FSLP framework. Therefore, researchers' primary model for analysis was multiple regression because the research's objective was to assess gendered differences in the attitude toward the FLSP framework among CRE teachers without considering the time effect. Before the multiple regression analysis, researchers examined data credibility for regression using pairwise Pearson's correlation between the independent variables and multicollinearity using variance inflation factors, and checked whether an explanatory variable is correlated with the error term using Durbin and Wu-Hausman test. Multiple regression was also preferred over other models because it assesses the four explanatory variables simultaneously instead of separately [ [82,83]; [ [84,85]]. Multiple regression modeling could analyze these four explanatory variables [ [86]; [ [[87], [88], [89]]].

The multivariate regression modeling is as described in Equation (4).

(4) $$Y=\beta_{0}+\beta_{1}X_{1}+\beta_{2}X_{2}+\beta_{3}\;X_{3}+\beta_{4}X_{4}+\mu$$

where Y is the attitude, β_0_ is the intercept, β_1_, β_2_, β_3_, and β_4_ are the regression coefficients of independent variables, X_1_ is the academic qualification, X_2_ is the teaching experience, X_3_ is the awareness, X_4_ is the utilization, and μ is the error term.

### 2.6 Ethical consideration

The processes involved undergoing a research ethics review and seeking informed consent from the National Council for Science and Technology in the Ministry of Higher Education, Science and Technology (NACOSTI). The researcher obtained a research license No: NACOSTI/P/21/13591 and approval letter from the County Commissioner and the Ministry of Education offices in Meru County. The participants voluntarily took part in the research after signing a consent form. The respondents were assured that the data would be analyzed and reported without revealing their identities.

## 3 Results and discussion

### 3.1 Descriptive characteristics

Table 1 displays the descriptive nature of the sampled CRE teachers. The CRE teachers' descriptive characteristics were categorized by gender-based on education, experience, awareness, and utilization of the FSLP lesson plan (Table 2). The study revealed that education, experience, awareness, and utilization did not differ significantly between the genders of the CRE teachers. However, two hundred and two participants (89.4%) were degree holders. Most males (90.4%) and females (88.8%) had a degree. The study findings agreed with [90], who indicated that many CRE instructors in secondary schools had a bachelor's degree in Nigeria. In Kenya, a diploma degree from a recognized institution is the minimum requirement to teach CRE at the secondary level [91].

**Table 1:** Variables description.

Variables	Explanation	Sign
Dependent variable		
Attitude	Categorical: 1 Strongly Disagree, 2 Disagree, 3 Undecided, 4 Agree, 5 Strongly Agree	
**Independent variables**		
Gender	Binary: 1 if female, 0 male	
Qualification	Categorical: 1 Diploma degree, 2°, and 3 masters	+
Experience	Categorical: Below 5, 6–10, 11–15, 16–20 and above 20	+
Awareness	Ordinal: Number of stages a teacher was aware of	±
Utilization	Ordinal: Number of stages a teacher implemented	±

**Table 2:** Demographic characteristics of the participants in Meru County.

Variables Categorical	Description	Pooled (N = 226)	Male (M), N = 83)	Female (M), N = 143)	P-Value Chi-square
Education	Diploma	8(3.5)	3(3.6)	5(3.5)	0.89ns
	Degree	202(89.4)	75(90.4)	127(88.8)	
	Postgraduate	16(7.1)	5(6.0)	11(7.7)	
Experience	5 years and below	98(43.4)	38(45.8)	60(42)	0.93ns
	6-10 7 years	76(33.6)	28(33.7)	48(33.6)	
	11–15 years	35(15.5)	11(13.3)	24(16.8)	
	16–20 years	7(3.1)	2(2.4)	5(3.5)	
	Above 20 years	10(4.4)	4(4.8)	6(4.2)	
**Continuous**	**Description**	**Pooled**	**Mean**	**Mean**	**P-value *t*-test**
Awareness	Number of stages a teacher knew	1.60	1.69	1.50	0.374ns
Utilization	Number of stages a teacher implemented	3.69	3.80	3.57	0.132ns

Parenthesis is the percentage, ns not statistically significant at 5% level of significance.

Study findings showed that most male (45.8%) and female (42%) CRE teachers had taught for five years and below. The pooled results showed that most teachers (43.6%) had 5 years of experience and below. The findings indicated that female participants had a higher experience than males. The results revealed more female CRE teachers than their male counterparts. These findings were in harmony with [34], who affirmed that most male partners perceive CRE as a subject for female learners when selecting learning areas. The similar findings were put forward by Ref. [22] who indicated that CRE is a noble subject directly connected to and enjoyed by females.

The mean implementation of the FSLP framework for males (3.80) and males (3.57) was statistically similar. The pooled average of 3.69 revealed that most CRE teachers were aware of and implemented more than 3 stages of the FSLP framework.

Regarding awareness of the FSLP framework, there were no significant differences between male and female CRE teachers (Table 2). The results revealed a mean awareness of 1.60. The male teachers had an average awareness of 1.69 and female of 1.50. The results suggested that all the participants knew at least one stage of the FSLP framework. The findings were supported by [ [92,93]] who found that lesson closure, the last stage of the FSLP framework, is highly utilized among teachers because it provides them with a platform to summarize the entire content. In contrary to the findings [67], indicated a significant difference between male and female teachers based on awareness of innovative teaching strategies such as the FSLP framework.

### 3.2 Perception of the FSLP framework

The plausibility of the constructs in determining the attitude towards the FLSP framework among CRE teachers was tested using Cronbach's Alpha coefficient method. The findings revealed a coefficient of 0.723. The coefficient was greater than 0.7; thus, items were reliable and plausible for the CRE teachers' attitudes determination [ [94,95]]. Researchers calculated the weighted average index (WAI) to rank attitudes towards the FSLP framework (Table 3). The results found that ' A teacher is self-assured when teaching CRE using FSLP’ was the most common positive construct among CRE teachers, with a WAI of 4.589. The result revealed that the overall WAI was 3.591, which is favorable; therefore, CRE teachers had a positive attitude towards this framework.

**Table 3:** The teachers' attitudes toward the FSLP framework.

Variables	Description	Pooled (N = 226)	Rank	Male (M), N = 83)	Rank	Female (F), N = 143)	Rank	Diff (M − F)
Confidence	A teacher is self-assured when teaching CRE using FSLP	4.58	1	4.55	1	4.60	1	−0.047ns
Platform	The FSLP provides the teacher with the best stage set for teaching	4.57	2	4.53	2	4.59	2	−0.057ns
Directives	The FSLP framework dictates the process of teaching CRE	4.25	3	4.14	3	4.31	3	−0.170ns
Preference	A teacher wishes to use the FSLP while teaching	3.74	4	3.73	4	3.75	4	0.013ns
Understanding	Current CRE content is comprehensible through FSLP	3.36	5	3.37	6	3.36	6	0.017ns
Application	A teacher can jointly implement the 5 stages of the FSLP	3.35	6	3.39	5	3.34	7	0.050ns
Relevance	FSLP is suitable for content delivery in CRE	3.41	7	3.33	7	3.46	5	−0.136ns
User-friendly	FSLP is simple and convenient	3.19	8	3.28	8	3.15	8	0.130ns
Training	A teacher was prepared on how to apply FSLP	2.11	9	2.01	9	2.17	9	−0.156ns
Average attitude		3.62		3.60		3.64		−0.043ns

Mean attitude, Ns mean the difference not significantly different at p<5%.

The rank highlighted some disparities between male and female teachers (Table 3). The CRE teachers indicated their level of agreement with the statement in Table 3. The findings indicated no significant differences between female and male teachers' attitudes toward the FSLP framework at a 5% significance level.

Regarding confidence, the pooled, male and female CRE teachers agreed (4.58, 4.55, and 4.60) that the FSLP framework they are self-assured when teaching CRE. The findings were in harmony with [ [96,97]], who indicated that lesson planning makes teachers confident. The results showed an insignificant mean difference (−0.047) between males and females. Considering the platform, the pooled, male and female teachers agreed (4.57, 4.53, and 4.59) that the FSLP framework provided the best stage set for teaching. The study revealed a negative but insignificant mean difference (−0.057) between males and females.

In terms of the directive, pooled male and female respondents agreed (4.25, 4.14, and 4.31) that the FSLP framework helped them guide learners during the learning process. The results implied a negative but insignificant mean difference between male and female participants (−0.170). Concerning preference, the pooled male and female participants established (3.74, 3.73, and 3.75) that they wished to utilize the FSLP framework while teaching. The study outlined a positive but insignificant mean difference between male and female teachers (0.013). Based on the understanding of the framework, participants agreed (3.36, 3.37, and 3.36) that the current CRE content is understandable through the FSLP framework. There was a positive but insignificant mean difference between male and female CRE teachers (0.017).

Regarding the framework's application, CRE teachers approved (3.35, 3.33, and 3.36) that they could implement the five stages of the framework jointly. There was a positive but insignificant mean difference between male and female CRE teachers (0.050). Concerning the relevancy of the framework, CRE teachers approved (3.41, 3.33, and 3.46) that the FSLP framework is suitable for content delivery in CRE. There was a negative but insignificant mean difference between male and female CRE teachers (−0.136).

Concerning the construct of user-friendly, CRE teachers approved (3.19, 3.28, and 3.15) that this item was one of the least in assessing their attitude. Few teachers established that the FSLP framework was simple and convenient. The study findings indicated insignificant mean differences (0.130) between male and female CRE teachers. Lastly, on the training item, participants indicated (2.11, 2.01, and 2.17) that it was the last construct in assessing their attitude towards the FSLP framework. Few teachers indicated that they were prepared to utilize the FSLP framework. Training was used as a control variable, where all the negative reactions, for instance, strongly disagree, disagree, and undecided were considered untrained (No).

In contrast, all the positive reactions, strongly agree and agree, were considered trained (Yes). The result suggested that most respondents claimed that they needed in-service training on the FSLP framework. The findings agreed with [98], who outlined in-service training as necessary to equip instructors with the essential skills to adopt the novel instructional framework. The results indicated insignificant mean differences (0.156) between male and female respondents.

Attitudinal change is a critical issue in the educational sector [99]. Christian Religious Education teachers generally had a positive attitude, which was favorable for males and females (3.62, 3.60, and 3.64) towards the FSLP framework, which was favorable. The result implied that the participants were ready to embrace the implementation of the framework. This finding corroborated with[ [64,100,101]], who found that teachers construct positive attitudes towards newly emerged strategies. There was a negative but insignificant mean difference between male and female CRE teachers (−0.043).

### 3.3 Relationship between genders of the CRE teachers and their attitude towards the FSLP framework

#### 3.3.1 Model summary

The R-squared is the coefficient of determination that describes how the independent variables explain the outcome [88]. Adjusted R Square is used as the coefficient of determination for regression analysis of many independent variables. The R squared were 0.062, 0.056, and 0.049 for overall male and female CRE teachers, respectively (Table 4). The results indicated the input of the effect of variables X_1_-X_4_ (utilization, experience, qualification, and awareness) to the dependent variable (gendered attitude on the five-stage lesson plan framework) of 6.2%, 5.6%, and 4.9%. The remaining 93.8%, 94.4%, and 95.1% are described by other variables not involved in our research model. The findings underscored the importance of gender on the attitude towards the FSLP framework among CRE teachers.

**Table 4:** Model summary.

Sample	R	R square	Adjusted R square	Std. Error of Estimate
Pooled	.281^a^	.079	.062	.483312
Male	.320^a^	.102	.056	.470901
Female	.276^a^	.076	.049	.495373

a Predictors: (Constant), Utilization, Experience, Qualification, Awareness.

#### 3.3.2 Analysis of variance (ANOVA)

Table 5 presents the ANOVA findings. The ANOVA tests the model goodness-of-fit [ [[102], [103], [104]]]. Researchers tested the null hypothesis that the fit of the intercept model is only equal to the study model. Based on the results, the p-values were significant (p < 0.001, p = 0.034, and p = 0.027) for pooled, male, and female respondents. Therefore, the study rejected the null hypothesis. Thus, the multiple regression model fits the data better than the intercept model.

**Table 5:** Analysis of Variance results.

Sample		Sum of squares	Df	Mean square	F	P-value.
Overall	Regression	4.409	4	1.102	4.718	.001^b^
	Residual	51.624	221	.234		
	Total	56.032	225			
Male	Regression	1.970	4	.592	2.221	.034^b^
	Residual	17.296	78	.222		
	Total	19.266	82			
Female	Regression	2.788	4	.697	2.840	.027^b^
	Residual	33.864	138	.245		
	Total	36.652	142			

a. Dependent Variable: Attitude.

#### 3.3.3 Linear regression findings

Table 6 presents the findings of the multiple regression. The study findings showed the influence between variables in determining the gendered differences in the attitude of the FSLP framework among CRE teachers. Similar determinants influence teachers' attitudes towards the FSLP framework. However, the regression result revealed that teachers' attitudes towards the FSLP framework did not significantly differ between male and female teachers. Gender in innovation is unseen because several kinds of research on innovation are based on processes or organizations and not gender [36]. Nevertheless, the existing studies focusing on innovation based on gender have shown innovation to be an extremely gendered field.

**Table 6:** Coefficient results.

Sample	Model	Unstandardized Coefficients		Standardized Coefficient	T	P-value
		B	Std Error	Beta		
Pooled	(Constant)	3.398	.244		13.929	0.000
	Qualification	−.034	.100	−.022	−.344	0.032
	Experience	0.062	.031	.026	2.000	0.043
	Awareness	.071	.019	.251	3.737	0.030
	Utilization	.059	.028	.072	2.107	0.027
Male	(Constant)	3.605	.244		13.929	0.000
	Qualification	−.234	.100	−.022	−.234	0.029
	Experience	.072	.031	.046	2.323	0.038
	Awareness	.081	.021	.251	3.838	0.001
	Utilization	.064	.031	.072	2.065	0.021
Female	(Constant)	3.311	.419		8.603	0.000
	Qualification	−.215	.168	−.074	1.280	0.046
	Experience	−.071	.050	−.025	1.420	0.022
	Awareness	.092	.033	.304	2.811	0.006
	Utilization	.063	.049	.050	1.286	0.046

a. Dependent Variable: Attitude.

Qualification negatively and significantly influenced the attitudes towards the FSLP framework for pooled male and female teachers at a 5% significance level. The regression coefficient for qualification were (**β** = −0.034, p = 0.032) for pooled, (**β** = −0.234, p = 0.029) for males and (**β** = −0.215, p = 0.046) for females. In agreement with the study results, it can be argued that an increase in academic qualification among the CRE teachers was connected to a decrease in a positive attitude towards the FSLP framework. In support of the study results, the triadic model of attitude formation theory as described by Ref. [44] indicates teachers’ qualification as a major predictor of their attitudes towards teaching methodology such as the FSLP framework. In support of the study findings, negative prediction of the academic qualification could be ascribed to various aspects not considered in this study, such as the time factor and age of the participants, as opposed to the gendered difference in attitude toward the FSLP framework. Contrary to the study findings [ [[105], [106], [107]]], teachers' qualification is an influential determinant of their attitude toward a novel teaching strategy.

Experience positively and significantly influenced the attitudes towards the FSLP framework for pooled male and female teachers at a 5% significance level. The regression coefficient for experience were **(β** = 0.062, p = 0.043) for pooled, **(β** = 0.072, p = 0.038) for males and (**β** = 0.071, p = 0.022) for females. The findings suggested that if the other independent variable values are fixed, each addition of the CRE teachers' experience increases the positive attitude towards the FSLP framework. In this regard, it can be argued that increasing CRE teachers' experience increases the positive attitude towards the FSLP framework. The study findings agreed with [ [108,109]], who found that teachers' experience was a key factor in predicting teachers' feelings and implementing any instructional strategy. Additionally, gender schema theory, as described by Ref. [42] outlines teachers’ experience as one of the key determinants of the attitudes towards any teaching methodology introduced to teachers.

Awareness positively and significantly influenced the attitudes towards the FSLP framework for pooled male and female teachers at a 5% significance level. The regression coefficient for the awareness were (β = 0.071, p = 0.030) for pooled, (β = 0.081, p = 0.001) for males and (β = 0.092, p = 0.006) for females. The findings indicated that if the other independent variable values are fixed, the awareness rate among CRE teachers increases the positive attitude towards the FSLP framework for each addition. Additionally, awareness was a strong positive predictor of the attitude towards the FSLP framework among CRE teachers. The regression results outlined that creating awareness among CRE teachers significantly influenced their attitude towards the FSLP framework. Based on the regression results, an increase in FSLP framework awareness among CRE teachers increases their attitude toward the framework. One of the study's theory, self-perfection theory, supported this study's findings by clarifying teachers' awareness and prior knowledge as vital components of attitude towards innovative or any novel teaching strategy. Furthermore, the study results were consistent with those [ [65,110,111]] who outlined that creating awareness of a teaching approach among teachers increases positive attitudes towards the same approach.

Utilization positively and significantly influenced the attitudes towards the FSLP framework for pooled male and female teachers at a 5% significance level. The regression coefficient was **(β** = 0.059, p = 0.027) for pooled, (**β** = 0.064, p = 0.021) for males and (**β** = 0.063, p = 0.046) for females. The results established that if the other separate variable values were fixed, each addition of the utilization of the framework would increase the rate of attitude towards the framework. In support of the regression results, increasing the utilization of the framework increases the positive attitude towards the framework among CRE teachers. A positive coefficient (+) means a positive relationship exists between the level of utilization and the attitude towards the framework; the more the framework utilization increases, the rate of attitude towards the FSLP framework. Utilizing the FSLP framework was a resilient positive predictor of the attitude towards the framework. The study results were in agreement with [[66,112]], who found that practicing lesson planning increases teachers' desire for the lesson preparation process. Contrary to the study's findings [23], found that lesson planning by teachers is viewed as a punishment and time wastage by this teachers. She described lesson planning a burden responsibility put on teachers.

Generally, this study investigated the gendered differences in the attitudes towards the five-stage lesson plan framework. It aimed to determine the gendered differences and assess the gendered determinants of attitude toward the FSLP framework among CRE teachers. The question investigated if there were any statistically significant gendered differences in attitude toward the FSLP framework. The study results discovered that being male or female is not a determining factor in shaping the CRE teachers' attitude towards the FSLP framework. This indicated that CRE teachers' attitudes towards the FSLP framework did not significantly differ based on gender. To support these findings [28], indicated that gender differences do not affect attitudes towards health information.

Contrary to our findings [27], defined gender as the main factor affecting the instructor's attitude in the teaching field. A study by Ref. [29] established that male teachers were less optimistic than their counterparts in their attitude toward the teaching profession. In support of the findings [30], confirmed a considerable gendered difference in teachers' attitudes towards their instructional practices.

Generally, our study findings indicated that irrespective of being male or female, CRE teachers had an overall WAI of 3.591, which is favorable. Therefore the participants had a positive attitude towards the FSLP framework. It indicated that attitude is an intense sensation that affects all CRE teachers.

The second research question explored whether there were any statistically significant gendered determinants of attitude toward the FSLP framework among CRE teachers in Meru County, Kenya. To answer this research question, the study employed multiple linear regression modeling. The regression findings documented and confirmed that the teacher’ attitudes towards the FSLP framework did not significantly differ between male and female CRE teachers. The teaching experience, utilization, awareness, and academic qualification were recognized as the gendered determinant factors which affected the attitude towards the FSLP framework among the CRE teachers.

The results indicated that the academic qualification was a strong negative predictor of the participants' attitude toward the FSLP framework. According to the results, it was acknowledged that academic qualification negatively and significantly influenced teachers' attitudes towards the FSLP framework. However, teaching experience, awareness, and utilization of the framework were strong positive determinants of the respondents' attitudes towards the framework.

## 4 Conclusion and policy recommendations

The study sought to empirically analyze gender differences in the attitudes toward the five-stage lesson plan framework amongst Christian Religious Education teachers in Meru County, Kenya. Researchers used, academic qualification, experience, awareness, and utilization of the FSLP framework as the attitudinal determinants. Further, the study found that Christian Religious Education teachers' attitudes toward the FSLP framework did not significantly differ based on the gender. The study findings also observed that teachers' academic qualification was negative and significant predictor of attitudes toward the FSLP framework among the CRE teachers. Additionally, teachers' experience, awareness and utilization of the FSLP framework were positive and significant predictors of attitudes toward the FSLP framework. The researchers, therefore conclude that teachers’ diversity in relation to academic qualification, awareness, utilization, and experience plays an important role in establishing gender differences in the attitudes toward the FSLP framework among teachers. This implies that the future researchers need to diversify their respondents in terms of academic qualification, utilization, awareness and experience to increase the chances of establishing gender differences in attitudes toward innovative strategies of teaching. In addition, ministry of education and the future researchers need to understand that gender is not a significant predictor of attitudes toward any innovative teaching strategies such as the FSLP framework.

The limitation of the study is that the FSLP is a novel teaching strategy among teachers and therefore getting data on it required a lot of time in the present study. The study's findings underscore the importance of teachers' diversity on establishing the attitudes toward any innovative teaching strategy. The findings imply that policymakers targeting to establish gender difference in the attitudes toward innovative teaching strategies should consider diversifying the respondents on the basis of qualification, awareness, utilization and experience. A longer time span study on gender differences in the attitudes toward the FSLP framework in other parts of Kenya can be studied in the future to provide more insights into application of the FSLP framework. A similar study can be done at different stages of the educational system, such as Primary schools, Colleges, and Universities, since the settings are dissimilar. Furthermore, the study proposes further research to jointly analyze female and male attitudes towards the FSLP framework using an appropriate model such as bivariate analysis. Lastly, future studies could consider assessing the influence of age diversity and job satisfaction on the attitudes toward the FSLP framework.

## Author contribution statement

Victor O. Saoke: Conceived and designed the experiments; Performed the experiments; Analyzed and interpreted the data; Contributed reagents, materials, analysis tools or data; Wrote the paper.Collins M. Musafiri: Conceived and designed the experiments; Analyzed and interpreted the data; Wrote the paper. Zachary N. Ndwiga: Conceived and designed the experiments; Performed the experiments; Wrote the paper. Pauline W. Githaiga: Conceived and designed the experiments; Contributed reagents, materials, analysis tools or data; Wrote the paper.

## Data availability statement

Data will be made available on request.

## Declaration of competing interest

The authors declare that they have no known competing financial interests or personal relationships that could have appeared to influence the work reported in this paper.
